# A long-term retrospective analysis of the haemorrhagic fever with renal syndrome epidemic from 2005 to 2021 in Jiangxi Province, China

**DOI:** 10.1038/s41598-023-29330-4

**Published:** 2023-02-08

**Authors:** Shiwen Liu, Tianchen Zhang

**Affiliations:** 1 Laboratory of Viral Infectious Disease, Jiangxi Provincial Center for Disease Control and Prevention, 555 Beijing East Road, Nanchang, 330029 China; 2 Emergency Office and Acute Infectious Disease Prevention Institute, Jiangxi Provincial Center for Disease Control and Prevention, Nanchang, 330029 China

**Keywords:** Infectious diseases, Kidney diseases

## Abstract

Jiangxi is one of the provinces in China most seriously affected by the haemorrhagic fever with renal syndrome (HFRS) epidemic. The aim of this paper was to systematically explore the HFRS epidemic in Jiangxi from the perspective of Hantavirus (HV) prevalence in rodents and humans and virus molecular characteristics. Individual information on all HFRS cases in Jiangxi from 2005 to 2021 was extracted from the China Information System for Disease Control and Prevention. All S and M fragment sequences of the Seoul virus and Hantan virus strains uploaded by Jiangxi and its neighbouring provinces and some representative sequences from provinces in China or some countries of Southeast Asia with the highest HV prevalence were retrieved and downloaded from NCBI GenBank. Periodogram and spatial autocorrelation were adopted for temporal periodicity and spatial clustering analysis of the HFRS epidemic. Joinpoint regression was utilized to explore the changing morbidity trend patterns of HFRS. Multiple sequence alignment and amino acid variation analysis were used to explore the homology and variation of strain prevalence in Jiangxi. Based on monthly morbidity time series, the periodogram analysis showed that the prevalence of HFRS had periodicities of 6 months and 12 months. Spatial autocorrelation analysis showed that HFRS distributed in Jiangxi was not random, with a “High-High” clustering area around Gaoan County. HFRS morbidity among the 0 ~ 15-year-old and ~ 61-year-old or older populations in Jiangxi increased significantly during the period of 2008–2015. Generally, HFRS morbidity was significantly positively correlated with the index of rat with virus (IRV) (r = 0.742) in the counties surrounding Gaoan from 2005 to 2019. HTNV strains in Jiangxi were in one independent branch, while the SEOV strains in Jiangxi were relatively more diverse. Both the YW89-15 and GAW30/2021 strains shared approximately 85% nucleotide homology and approximately 97% amino acid homology with their corresponding standard strains and vaccine strains. GAW30/2021 and YW89-15 had some amino acid site variations in nucleoprotein, glycoprotein precursor and RNA-dependent polymerase with their corresponding vaccine strains Z10 (HTNV) and Z37 (SEOV). The HFRS epidemic in Jiangxi has obvious temporal periodicity and spatial clustering, and the significant increase in the non-Immunization Expanded Program (EPI) targeted population (children and elderly) suggests that HFRS vaccination in this population needs to be considered. Although applying the EPI played a certain role in curbing the incidence of HFRS in Jiangxi from the perspective of ecological epidemiology, HTNV and SEOV strains prevalent in Jiangxi have some amino acid site variations compared to their corresponding vaccine strains, suggesting that HV variation needs to be continuously monitored in the future to observe vaccine protective efficiency.

## Introduction

Hantaviruses (HVs) are a genus of single-stranded, enveloped, negative-sense RNA viruses that usually cause chronic asymptomatic infection in rodents^1^. The genome of HV includes large (L), medium (M) and small (S) segments, which encode viral RNA polymerase, envelope glycoproteins Gn and Gc, and nuclear protein (NP), respectively^2^. The L segment is the most conserved among the three segments; the M segment expresses Gn and Gc proteins, which have neutralizing antigen sites and haemagglutination active sites that can induce the production of neutralizing antibodies and haemagglutination-inhibiting antibodies; and the NP encoded by the S segment can stimulate the humoral and cellular immune responses of the body^3^. Regarding the relationship between HV and natural hosts, the ecological environment and evolution, scholars have found that the epidemic of HV and the evolution of the epidemic area are the result of the long-term interaction between the virus and the host^4^. The geographical distribution and natural history of natural hosts play a key role in the prevalence range and intensity of HV-related diseases^5^.

When humans are incidentally infected with some strains of HV through inhalation of virus-containing aerosols or being bitten by rodents, two human syndromes can result: HV cardiopulmonary syndrome (HCPS) in the Americas and haemorrhagic fever with renal syndrome (HFRS) in Asia^6^. HFRS is a group of rodent infectious diseases with typical clinical manifestations of fever, haemorrhage, headache, abdominal pain and acute kidney damage that varies from mild to severe and is related to different pathogens^7^. In Europe, the primary HFRS pathogens are Puumala virus (PUUV) and Dobrava-Belgrade virus (DOBV), while in Asia, the primary pathogens are Hantan virus (HTNV), Amur virus (AMRV) and Seoul virus (SEOV)^8^.

Suspected HFRS records might be traced back to the 1930s, but it did not attract substantial attention until more than 3000 United Nations soldiers were infected from 1950 to 1953 in the Korean War^9^. In 1978, Lee discovered that its causative agent was HTNV, and the natural reservoir was the striped field mouse, *Apodemus agrarius*^10^. Although the understanding of HV infection has increased considerably worldwide over the past few decades, both the amplitude and the magnitude of HV outbreaks have been increasing, and several novel HVs with unknown pathogenicity have been identified in some insectivore hosts^11^.

It is estimated that more than 20000 HFRS cases occur every year globally, with the majority occurring in Asia. Since the first clinical HFRS case was detected in mainland China in 1931, China has gradually become the most prevalent country in the world, with 209209 HFRS cases and 1855 deaths reported during 2004-2019, accounting for more than 90% of the cases worldwide^12,13^. In Jiangxi, the first HFRS case was identified in Pengze County in 1961; since then, the number of epidemic counties and HFRS cases has gradually increased^14^. From 2005 to 2010, the reported annual incidence of HFRS in Jiangxi was maintained at approximately 1/100000 and then gradually increased to 1.5/100000, which has brought a serious economic burden to society and individuals. In 2009, Jiangxi carried out the HFRS-targeted Immunization Expanded Program among people aged 16-60 years in three high-incidence counties^15^.

Although several studies have described the epidemiological characteristics in some provinces of China^16–19^, no research has systematically explored the HFRS epidemic trend from the perspective of HV prevalence in rodents and humans and virus molecular characteristics. The aim of this research was to multidimensionally describe the characteristics of the HFRS epidemic in Jiangxi from 2005 to 2021 in terms of the temporal and spatial characteristics, the relationship between rats and the human epidemic, and the pathogenetic variation of HV.

## Methods

### Data resource and collection

**Figure 1:**
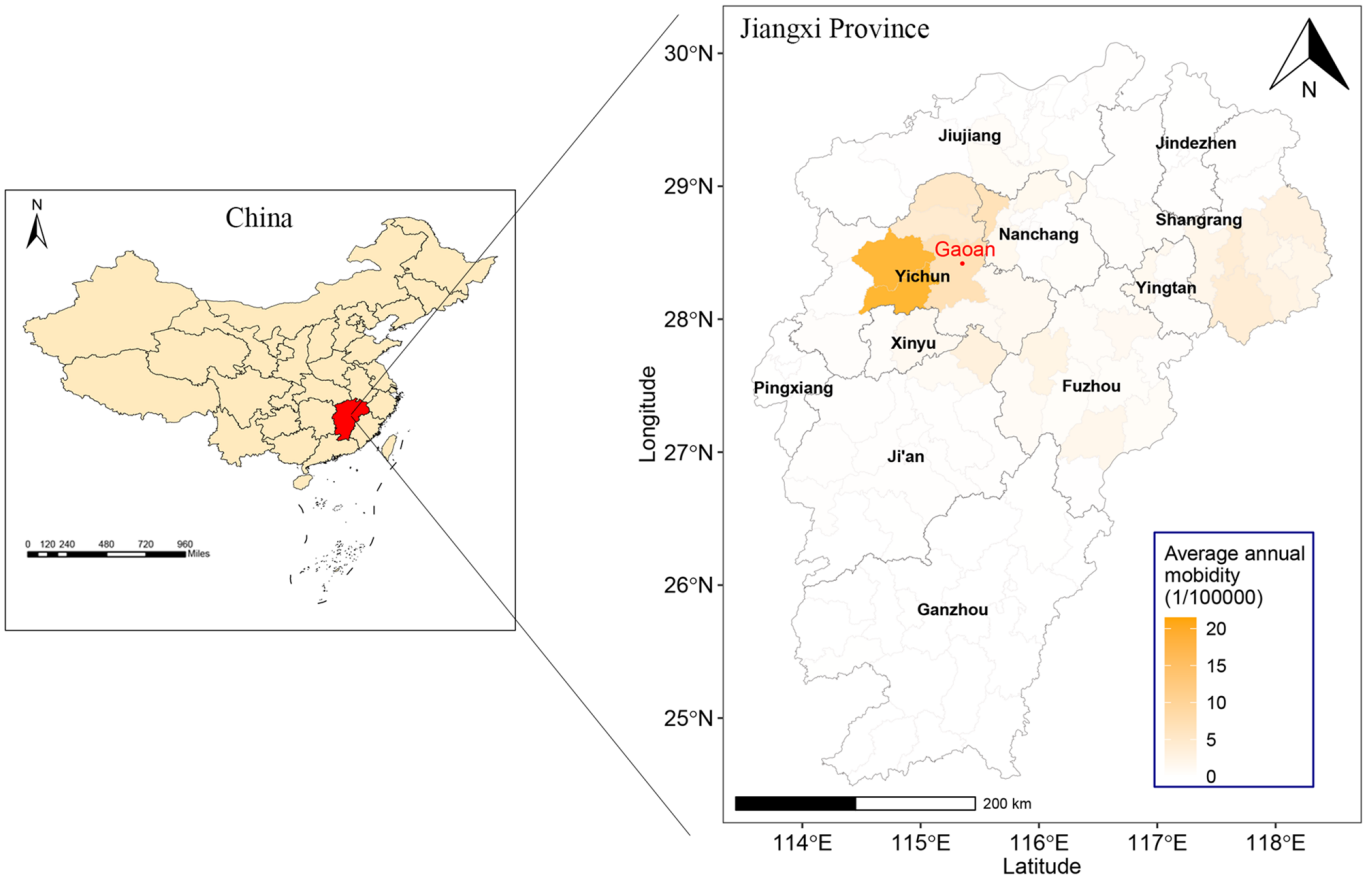
Location of Jiangxi in China and the HFRS morbidity distribution. The map was generated by ArcGIS software (Version 10.4 ESRI, Redlands, CA, USA, https://www.esri.com/sofware/arcgis/arcgis-for-desktop).

Jiangxi is located in south eastern China (24°29′-30°04′N, 113°34′-118°28′ E), a humid subtropical climate area that includes 11 cities and 100 counties (Fig. 1). The individual information of all reported HFRS cases in Jiangxi from 2005 to 2021, including demographic information (age, sex, occupation, residential address), diagnostic types (laboratory confirmed or clinical diagnostic cases; the diagnostic criteria are shown in text 1 of Supplementary Material 1), and dates of illness onset and diagnosis were obtained from the China Information System for Disease Control and Prevention (CISDCP), a web-based surveillance system to report and monitor infectious diseases used in China since 2004^15^. The population data at the county level were derived from the Basic Information System, a subsystem of the CISDCP. Geographic information data were extracted from the map of China (Approval Number: (2019) 1822). To explore the homology and variation of the epidemic strains between Jiangxi and the surrounding provinces, we retrieved and downloaded all S and M fragment sequences of Seoul virus (SEOV) and Hantan virus (HTNV) uploaded by neighbouring provinces of Jiangxi (Hunan, Hubei, Anhui, Zhejiang, Fujian and Guangdong Provinces) and other representative reference strains from NCBI GenBank (https://www.ncbi.nlm.nih.gov/genbank/).

### Rodent surveillance

HV surveillance in rodents was carried out by the night trapping method in spring and autumn seasons every year. Snap traps were set up in both field and residential areas to estimate the proportion of different species of reservoir rodents and the intensity of rodents. A direct immune fluorescent assay was used to detect HV-specific antigens in rodent lungs, and then the rodent density, positive number (PA), positive rate (PR), and index of rat with virus (IRV) were calculated to assess the HV epidemic intensity in rodents.

### Data analysis

Classification data are expressed as rates, and continuous data are expressed as the mean ± standard deviation or interquartile range (*IQR*) to reflect the concentration discrete trend. The temporal periodicity and spatial clustering of HFRS morbidity in Jiangxi from 2005 to 2021 were explored by periodogram based on Fourier transformation and spatial autocorrelation analysis determined by Moran’s *I* indicator using GeoDa software (Version 1.16), respectively^20,21^. Joinpoint regression was conducted by Joinpoint software (Version 4.9.1.0) to describe the time trend changing pattern of HFRS morbidity^22^. The join points were identified by the grid search method when linear trends changed significantly in direction or magnitude, and each trend change in the final model was described by an annual percentage change (APC) and average annual percentage change (AAPC) with 95% *CI*^23^. The multiple sequence alignment and amino acid variation analysis of the sequences included in this research were applied by MEGA X software (Version 11.0), and the neighbour-joining (NJ) method was used to construct the phylogenetic tree with a bootstrap test of 1000 replicates. Then, BioEdit software (Version 7.0) was used to conduct homology analysis. RDP4 (Version 4) and SimPlot software (Version 2.5) were used to conduct gene recombination signal analysis for the whole variant strain sequence to understand the variation in epidemic strains and vaccine strains.

### Ethics approval

All experimental protocols were approved by the Jiangxi Center for Disease Control and Prevention Ethics Committee. The data obtained from the CISDCP and GenBank were anonymized so that subjects could not be identified. The ethics committee of Jiangxi Center for Disease Control and Prevention waived the need for informed consent from patients due to the retrospective nature of the study. All methods in our study were used in accordance with the relevant guidelines and regulations. All animal experiments were reported in accordance with ARRIVE guidelines (Supplementary Material 2).

## Results

### Epidemic of human HFRS in Jiangxi

#### Distribution of HFRS cases

A total of 8981 HFRS cases were reported in Jiangxi between 2005 and 2021, with a case fatality rate (CFR) of 1.16% (104/8981). The median age was 47 years (*Q1* = 34, *Q3* = 58) with a sex ratio of 2.11:1 (male *vs.* female: 6093:2888). The occupation of patients was mainly farmers (5666), accounting for 63%, followed by students (720, 8%) and houseworkers (714, 8%).

#### Spatiotemporal distribution

**Figure 2:**
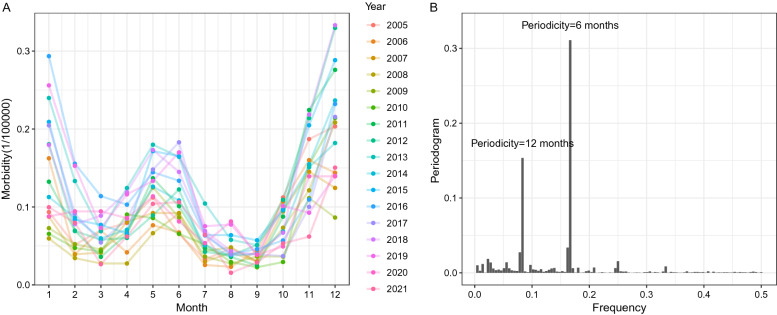
Monthly distribution and the periodogram of HFRS morbidity in Jiangxi from 2005 to 2021.

**Figure 3:**
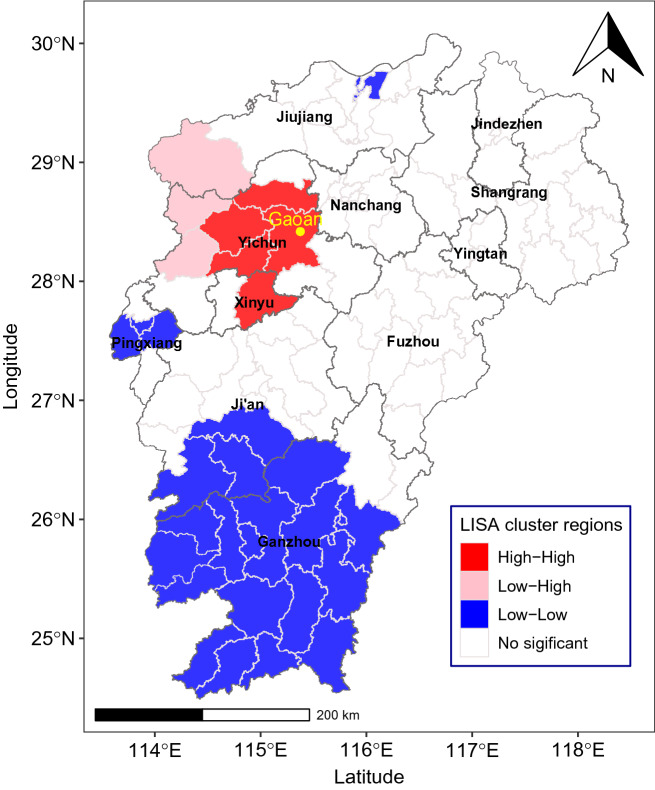
The local indicators of spatial association (LISA) cluster map of HFRS morbidity in Jiangxi Province from 2005 to 2021. The map was generated by ArcGIS software (Version 10.4 ESRI, Redlands, CA, USA, https://www.esri.com/sofware/arcgis/arcgis-for-desktop).

Figure 2 was drawn based on the monthly morbidity time series, which showed that the prevalence of HFRS had distinct peaks in winter and summer every year (Fig. 2A). Furthermore, the periodogram showed two significantly higher spikes at approximate frequencies of 0.166 and 0.083 Hz, which means that HFRS morbidity existed at 6 months (1/0.166) and 12 months (1/0.083) on the time-series scale (Fig. 2B). Spatial autocorrelation analysis found that the average annual morbidity of HFRS was not randomly distributed in Jiangxi from 2005 to 2021 (Moran’s I = 0.327, *P* = 0.002), with a “High-High” clustering area around Gaoan County and a main “Low-Low” clustering area in southern Jiangxi (Fig. 3).

#### Trend in HFRS morbidity

**Figure 4:**
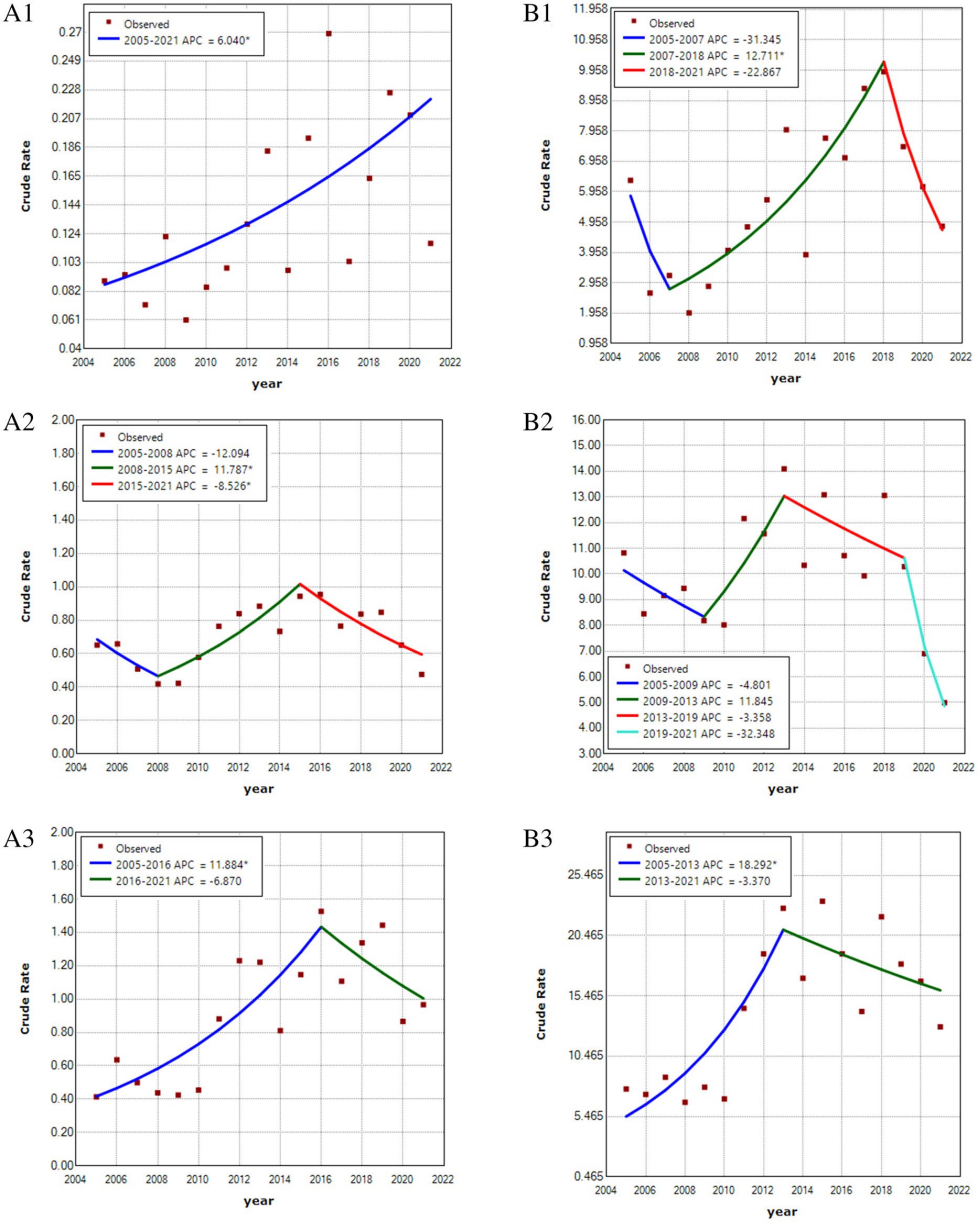
The trends in HFRS morbidity among the three age groups in high- and low-prevalence areas. A represents low prevalence areas and B represents high prevalence areas; 1, 2 and 3 represent 0 ~ 15 yrs, 16 ~ 60 yrs and ~ 61 yrs and older, respectively.

The trend of HFRS morbidity reported in Jiangxi demonstrated four stages, but the APC in each stage was not significant, and the annual incidence rate was stable at 0.8~1.6/100000 (AAPC = −1.4, 95% CI = −9.1 to −6.9, *P* =0.729) (Table 1, Fig. 1 in Supplementary Materials 1). The changes in HFRS morbidity trends among three age groups (0~15 yrs, 16~60 yrs and ~61 yrs and older) in high- and low-prevalence areas were further explored (an average annual morbidity greater than 5/100000 was defined as a high-prevalence area). As Table 1 and Fig. 4 show, the HFRS morbidity in the 0~15 yrs group significantly increased by an average of 6% per year from 2005 to 2021 (APC=6.0, *P* <0.05) in the low prevalence area and by 12% per year from 2007 to 2018 (APC=12.0, *P* <0.05) in the high prevalence area. The HFRS morbidity at 16~60 yrs (HFRS vaccine-targeted population) showed a significant increase of 11% per year from 2008 to 2015 (APC=11.0, *P* <0.05) in the low prevalence area, while no significant change was found in the high prevalence area. For ~61 yrs and older, the HFRS morbidity markedly increased by 11.8% per year from 2005 to 2016 in the low prevalence area (APC=11.8, *P* <0.05) and by 18.3% per year from 2005 to 2013 in the high prevalence area (APC=18.3, *P* <0.05).

**Table 1:** Morbidity trend by age according to the join points identified by the analysis.

Area	Age group (Yrs)	Period	APC (95% CI)	*P* value	AAPC (95% CI)	*P* value
All counties	Overall	2005 ~ 2007	− 9.6 (− 49.6, 61.8)	0.703	− 1.4 (− 9.1, 6.9)	0.729
		2007 ~ 2018	5.9 (1.4, 10.6) *	0.015		
		2018 ~ 2021	− 19.7 (− 38.9, 5.4)	0.101		
Low morbidity area	0 ~ 15 yrs	2005 ~ 2021	6.0 (2.0, 10.2)	0.005	6.0 (2.0, 10.2) *	0.005
	16 ~ 60 yrs	2005 ~ 2008	− 12.1 (− 3.9, 16.9)	0.333	− 0.9 (− 7.1, 5.8)	
		2008 ~ 2015	11.8 (1.8, 22.7) *	0.024		
		2015 ~ 2021	− 8.5 (− 16.2, − 0.2) *	0.046		
	~ 61 yrs and older	2005 ~ 2016	11.9 (4.9, 19.3) *	0.003	5.6 (− 0.2, 11.8)	0.057
		2016 ~ 2021	− 6.9 (− 19.3, 7.5)	0.301		
High morbidity area	0 ~ 15 yrs	2005 ~ 2007	− 31.3 (− 72.7, 72.3)	0.379	− 1.3 (− 12.8, 11.6)	0.381
		2007 ~ 2018	12.7 (5.7, 20.2) *	0.002		
		2018 ~ 2021	− 22.9 (− 47.2, 12.7 )	0.155		
	16 ~ 60 yrs	2005 ~ 2009	− 4.8 (− 19.1, 12.0 )	0.487	− 4.5 (− 13.6, 5.5)	0.366
		2009 ~ 2013	11.8 (− 14.2, 45.9 )	0.342		
		2013 ~ 2019	− 3.4 ( − 13.5, 9.0)	0.481		
		2013 ~ 2021	− 32.3 ( − 66.5, 36.6)	0.222		
	~ 61 yrs and older	2005 ~ 2013	18.3 (7.8, 29.8 ) *	0.002	6.9 (1.6, 12,5) *	0.010
		2013 ~ 2021	− 3.4 ( − 9.3, 2.9)	0.263

### Relationship between HV prevalence in rodents and the HFRS epidemic in humans

#### Rodent HV prevalence

A total of 1382200 mousetraps were set out in Gaoan County, one of the monitoring sites in China, and 3933 rats were captured, with an average rat density of 2.80% and an average annual antigen-positive rate of 2.01%. The average HV antigen-positive rate of rodents was 2.24% in fields, and the main species of rodents were *Rattus losea* (915/1957, 47%) and *Apodemus agrarius* (873/1957, 45%). The average HV antigen-positive rate of rodents was 1.79% in residential areas, and the main species of rodents were *Rattus norvegicus* (1679/1976, 85%) and *Mus musculus* (255/1957, 13%). Based on the rat density and antigen-positive rate, we calculated the index of rat with virus (IRV) to reflect the HV prevalence intensity among rodents. In general, the IRV showed a significant upwards trend from 2005 to 2013 and then remained relatively stable at approximately 2.5 in the next 8 years (Tables 1 and 2 in Supplementary Material 1 and Fig. 2 in Supplementary Material 1).

**Figure 5:**
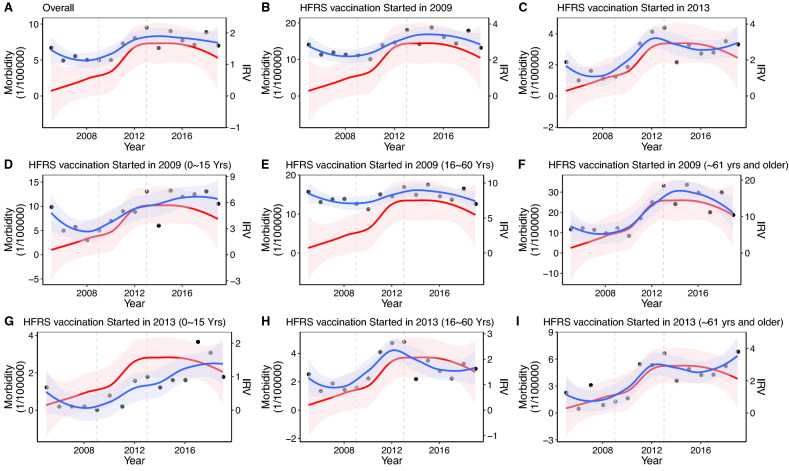
Correlation between human and rodent hantavirus from 2005 to 2021 in the counties surrounding Gaoan County. (**A**) shows the overall counties surrounding Gaoan County. (**B**, **D**, **E** and **F**) show the overall, 0 ~ 15, 16 ~ 60 and ~ 61 yrs and older subgroups in the counties that started HFRS vaccination in 2009, respectively. C, G, H and I show the overall, 0 ~ 15, 16 ~ 60 and ~ 61 yrs and older subgroups in the counties that started HFRS vaccination in 2013. The blue line is the HFRS morbidity fitted curve by loess regression, and the yellow line is the HFRS morbidity fitted curve by loess regression; the dots indicated the HFRS morbidity.

**Table 2:** Epidemiological correlation between human and rodent hantavirus from 2005 to 2019 in the surrounding counties of Gaoan.

Age (Yrs)	All Counties		Counties started in 2009		Counties started in 2013	
	r (95%CI) *	*P* value	r (95%CI) *	*P* value	r (95%CI) *	*P* value
Overall	0.706 [0.242, 0.880]	0.003	0.662 [0.227, 0.877]	0.007	0.688 [0.271, 0.887]	0.004
0 ~ 15 yrs	0.598 [0.122, 0.841]	0.018	0.555 [0.060, 0.831]	0.031	0.564 [0.073, 0.835]	0.028
16 ~ 60 yrs	0.600 [0.128, 0.851]	0.040	0.546 [0.047, 0.827]	0.035	0.586 [0.105, 0.844]	0.021
~ 61 yrs and older	0.742 [0.316, 0.897]	0.002	0.687 [0.271, 0.887]	0.004	0.723 [0.336, 0.901]	0.002

*Correlation coefficient was calculated based on Person test.

#### Relationship between IRV and HFRS morbidity

Due to the strict nonpharmaceutical interventions for coronavirus disease 2019 (COVID-19), the morbidity of many infectious diseases has declined from 2020 to 2021, including HFRS^24^. Therefore, this research only explored the correlation between HFRS morbidity and IRV in the 7 surrounding counties of Gaoan County from 2005 to 2019. As Fig. 5 and Table 2 show, IRV and HFRS morbidity presented a strong positive correlation (r=0.706, 95% CI: 0.242–0.880, *P* =0.003) in the overall population, especially in the ~61 yrs and older age group (r=0.742, 95% CI: 0.316–0.897, *P* =0.002). Furthermore, this strong positive correlation was found in every age subgroup in two areas with different vaccination start years. Due to the potential effect of vaccine intervention, from 2009 to 2013, the morbidity increased rapidly in most age subgroups, while the morbidity of the 16~60-year-old population in the region that started vaccination in 2009 was relatively stable (Fig. 5E). During the period from 2013 to 2018, when IRV was relatively stable, the morbidity of 16~60-year-olds showed a declining trend in the region that started vaccination in 2013 (Fig. 5H).

### Genetic variation of HV in Jiangxi

A total of 23 S and 34 M sequences isolated from Jiangxi were obtained from GenBank from 1986 to 2021, involving 26 strains belonging to HTNV and 8 strains belonging to SEOV. Among them, 11 strains were from human specimens, and 23 strains were from rodent specimens (Table 3 in Supplementary Material 1). All available HV strains in the surrounding provinces of Jiangxi and other representative reference sequences were also retrieved and downloaded from GenBank (Table 4 in Supplementary Material 1).

#### Phylogenetic analysis

**Figure 6:**
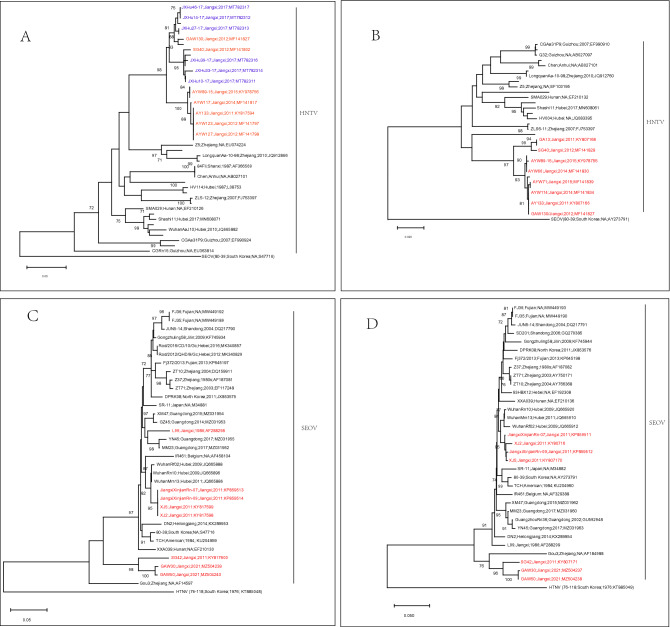
Genetic phylogenetic tree of hantavirus in Jiangxi and its surrounding provinces. (**A**): HTNV M gene; (**B**): HTNV S gene; (**C**): SEOV M gene; (**D**): SEOV S gene. The blue font indicates the virus strains isolated from human specimens in Jiangxi; the red font indicates the virus strains isolated from rodent specimens in Jiangxi.

As shown in Fig. 6A, the M sequences of HTNV strains isolated from Jiangxi in different years were all clustered in one independent branch, regardless of human or rodent specimens, which were mostly close to the Z5 and Z10 strains from Zhejiang Province. The S sequences of HTNV in Jiangxi were also in an independent branch, similar to the M sequences of HTNV (Fig. 6B). The SEOV strains in Jiangxi were relatively more diverse than HTNV and were distributed in three branches on the phylogenetic tree for both the M and S sequences, and the genetic relationship was closest to the isolates from Guangdong, Zhejiang and Hubei provinces (Fig. 6C and D).

#### Homology analysis

The nucleotide homology of HTNV M and S sequences among Jiangxi isolates were both approximately 95%, while they were only 77%~89% and 83%~87% compared to domestic and foreign reference strains, respectively. The nucleotide homology of SEOV M sequences among Jiangxi isolates was 84%~100% and was 85% - 97% compared to domestic and foreign reference strains. The nucleotide homology of SEOV S sequences among Jiangxi isolates was 89%~100% and was 87%~99% compared to domestic and foreign reference strains. However, whether HTNV or SEOV, the deduced amino acid sequence homology of the M and S sequences was more than 95% among Jiangxi isolates or compared to domestic and foreign reference strains.

#### Mutation analysis

The AYW89-15 strain and GAW30/2021 strain were selected as independent representative strains for HTNV and SEOV, respectively, and their gene sequences were compared with the standard strain and the HFRS bivalent inactivated vaccine strain. Compared with their corresponding standard strains, HTNV 76-118 and SEOV 80-39, the AYW89-15 and GAW30/2021 strains shared approximately 85% nucleotide homology and 97% amino acid homology. Compared with the vaccine strain HTNV Z10, the AYW89-15 strain shared approximately 83%~86% nucleotide homology and 95%~98% amino acid homology. Compared with the vaccine strain SEOV Z37, the GAW30/2021 strain shared approximately 84%~88% nucleotide homology and 97%~99% amino acid homology (Tables 5 and 6 in Supplementary Material 1). Compared to the vaccine strain HTNV Z10, AYW89-15 had 10, 38 and 57 amino acid site variations in nucleoprotein, glycoprotein precursor and RNA-dependent polymerase, respectively. Compared to vaccine strain SEOV Z37, GAW30/2021 had 5, 33 and 54 amino acid site variations in nucleoprotein, glycoprotein precursor and RNA-dependent polymerase, respectively (Supplementary Material 2).

## Discussion

The morbidity of HFRS in Jiangxi showed a rising trend after 2009 and then stabilized at 1.2~1.6/100000 from 2013 to 2019, which was similar to the national epidemic trend^16,25^. The relatively lower epidemic morbidity of HFRS in 2020 and 2021 might be affected by the strict control measures for COVID-19^24^. Consistent with other studies, this paper found two peaks of the HFRS epidemic in spring and winter in Jiangxi, which was mainly related to high contact rates linked to rodents’ indoor foraging activity and agricultural activities during the harvest season^18,26^. Based on the periodogram analysis, this study again verified that HFRS has semiannual and one-year periodic fluctuations, which was consistent with the results of Zou’s research using wavelet tools^13^. Previous studies found that some meteorological factors determined the living environment and habits of rodents and humans, which potentially contributed to the seasonal periodicity of HFRS^27–33^.

Based on the spatial autocorrelation analysis, this study found that the prevalence of HFRS in Jiangxi exhibited spatial clustering and regionality. Previous studies in Hunan and Hubei found that the prevalence of HFRS was limited to areas below 200 metres above sea level^34,35^, which might explain why HFRS showed “low-low” clustering in the southern mountainous areas of Jiangxi, where the altitude is mostly 300–500 metres^14^. Additionally, “High-High” areas were mainly distributed in the northeastern Poyang Lake Ecological Economic Zone, which is historically rich in fish and rice and more conducive to the survival of rodents^36^.

Similar to other studies, the results of this paper suggested a strong correlation of HV prevalence among rodents and the HFRS epidemic in humans^37^. From 2005 to 2016, the prevalence of HV among rodents presented a rapid increase in Jiangxi, which explained the increasing trend of the HFRS epidemic in humans during that period. At the same time, this study also noted the increasing trend of the nonvaccination-targeted population in both high- and low-incidence areas at an average annual growth rate of more than 10%, especially among the ~61 yrs and older population in high-incidence areas (18%), which was consistent with other studies^14,38^. The reasons for this finding could be attributed to three potential factors: first, due to the tremendous number of young people leaving villages for better work, elderly people and children must engage in more agricultural activities that increase their exposure risk^39^; second, accelerated urbanization changed the rodent living environment, which promoted contact between humans and rodents and accelerated the spread of HV^40^; and finally, HFRS immunization was only carried out among people aged 16–60 years in Jiangxi^41^. As this paper found, although the prevalence of HV in rodents increased or remained stable from 2009 to 2019, the morbidity among vaccination-targeted people remained at a certain level or decreased, which reflects the effectiveness of the vaccine from the perspective of ecological epidemiology.

Jiangxi is a mixed epidemic area containing two dominant specific hosts *of A. agrarius* and *R. norvegicus* with two coprevalent kinds of HV (HTNV and SEOV), which is a possible reason for a bimodal distribution of HFRS morbidity^40^. HTNV in Jiangxi was highly homologous and formed a relatively independent branch that was not closely related to HTNV viruses at home and abroad, suggesting that HTNV in Jiangxi may be a new subtype and may have geographical limitations. Unlike HTNV, there were multiple branches of SEOV in Jiangxi, and these genetic relationships were closely related to Guangdong, Hubei and Zhejiang provinces around Jiangxi, indicating the diversity of HV and the dynamics of virus variation in Jiangxi. This phenomenon may be related to the fact that the main host of SEOV, *R. norvegicus,* a domesticated rodent, is more likely to migrate with human activities^42^.

The results of this paper indicated that the Immunization Expanded Program played a certain role in curbing the incidence of HFRS in Jiangxi from the perspective of ecological epidemiology; however, the vaccine strains Z10 (HTNV) and Z37 (SEOV) were isolated in the 1980s from Zhejiang Province^43^, and their protective effect against current strains in Jiangxi still needs further monitoring and analysis. The HTNV strains prevalent in Jiangxi were in an independent branch, and the genetic distance was relatively far from Z10 with approximately 3% amino acid heterogeneity. Thus, the protective effect of the vaccine strain Z10 used in Jiangxi needs further evaluation from serological and epidemiological perspectives.

The current study has several limitations. First, due to the lack of a specific research design, this paper did not find direct evidence that vaccines could reduce the prevalence of HFRS. Second, this study did not distinguish HTNV strain-type and SEOV strain-type in human cases, so the direct relationship between the HV epidemic and HFRS morbidity needs further research with laboratory evidence. Last, although we observed that the S and M sequences of HTNV in Jiangxi were in an independent branch compared to other regions, we could not determine that this distinction between the sequences of Jiangxi and other regions was caused by temporal or spatial factors because the collection time of many of the reference sequences was unknown and the compared fragments of HV sequences collected after 2010 were not matched in this study.

## Conclusion

The prevalence of HFRS in Jiangxi has obvious temporal periodicity and spatial clustering. Morbidity among children and elderly individuals had a rapid upwards trend with the increasing prevalence of HV in rodents, indicating that it is urgent to accelerate the implementation of expanded immunization in this population. HTNV isolated in humans or rodents in Jiangxi is relatively genetically independent, while SEOV in Jiangxi presents much more diversity. Although the amino acid homology of both HTNV and SEOV prevalent in Jiangxi with the vaccine strains remained relatively high, HV variation still needs to be continuously monitored in the future to determine vaccine protective effectivity due to the dozens of amino acid site variations.

## Supplementary Information

Supplementary Information 1.

Supplementary Information 2.

Supplementary Information 3.

## Data Availability

The datasets generated and/or analysed during the current study are not publicly available due to a large amount of personal privacy but are available from the corresponding author on reasonable request.
