# Supplementary material for: A long-term retrospective analysis of the haemorrhagic fever with renal syndrome epidemic from 2005 to 2021 in Jiangxi Province, China
Source: Sci Rep. 2023 Feb 8;13:2268. doi: 10.1038/s41598-023-29330-4 (PMC9907874; doi:10.1038/s41598-023-29330-4)
Supplement: Supplementary file 1 — Supplementary Information 1. [file 41598_2023_29330_MOESM1_ESM.docx]

**SUPPLEMENTARY MATERIAL 1**

**Text 1. The diagnostic criteria of HFRS cases**

HFRS cases were classified as suspected, clinically diagnostic or confirmed cases according to the health industry standard of the People’s Republic of China for diagnostic criteria of HFRS. Briefly, 1) a patient with epidemiological history and clinical manifestations of fever or gastrointestinal symptoms was defined as a suspected case; 2) a suspected case with hypotension, renal function impairment, increased peripheral blood cell counts and thrombocytopenia, and positive urine protein was defined as a clinically diagnosed case; and (3) a clinically diagnosed case with one or more of the following criteria was defined as a confirmed case: positive serum-specific IgM antibody; specific IgG antibody 4 times higher than that in the acute phase; and Hantavirus RNA is positive or Hantavirus is isolated.

**Figure 1. General trend of HFRS morbidity in Jiangxi from 2005 to 2021**


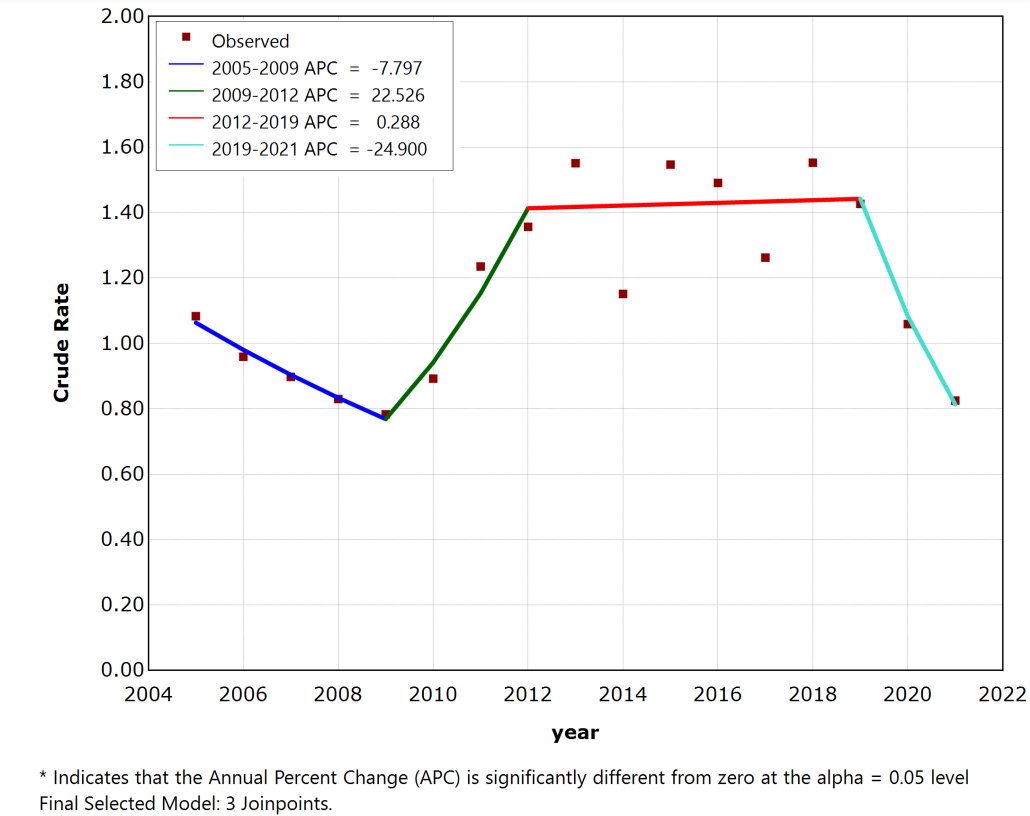


**Table 1. Rodent Hantavirus surveillances in Gaoan county from 2005 to 2021.**

| Year | Mousetraps | Rats captured | Rats’ density (%) | Rats detected | ANP (N) | APA (%) | IRV |
| --- | --- | --- | --- | --- | --- | --- | --- |
| 2005 | 10600 | 307 | 2.90 | 307 | 0 | 0.00 | 0.00 |
| 2006 | 5700 | 219 | 3.84 | 213 | 0 | 0.00 | 0.00 |
| 2007 | 6800 | 231 | 3.40 | 231 | 4 | 1.73 | 2.42 |
| 2008 | 8000 | 210 | 2.63 | 210 | 1 | 0.48 | 1.12 |
| 2009 | 10400 | 232 | 2.23 | 232 | 0 | 0.00 | 0.00 |
| 2010 | 11100 | 226 | 2.04 | 226 | 1 | 0.44 | 0.95 |
| 2011 | 12000 | 210 | 1.75 | 210 | 9 | 4.29 | 2.74 |
| 2012 | 9600 | 254 | 2.65 | 254 | 8 | 3.15 | 2.89 |
| 2013 | 9600 | 213 | 2.22 | 213 | 7 | 3.29 | 2.70 |
| 2014 | 9600 | 225 | 2.34 | 225 | 8 | 3.56 | 2.89 |
| 2015 | 9600 | 204 | 2.13 | 204 | 13 | 6.37 | 3.68 |
| 2016 | 9600 | 201 | 2.09 | 201 | 2 | 1.00 | 1.44 |
| 2017 | 6400 | 215 | 3.36 | 215 | 7 | 3.26 | 3.31 |
| 2018 | 5400 | 275 | 5.09 | 275 | 5 | 1.82 | 3.04 |
| 2019 | 3610 | 235 | 6.51 | 221 | 1 | 0.45 | 1.72 |
| 2020 | 3540 | 240 | 6.78 | 218 | 3 | 1.38 | 3.05 |
| 2021 | 6670 | 236 | 3.54 | 222 | 9 | 4.05 | 3.79 |
| Total | 138220 | 3933 | 2.85 | 3877 | 78 | 2.01 | 2.39 |

APN: Antigen positive number; APA: Antigen positive rate; Density= Rats captured/ Mousetraps*100%;

IRV: index of rat with virus =√(Density* APA)

**Table 2. Summary of Rodents distribution in Gaoan county from 2005 to 2021.**

| **Area** | **Species** | **Mousetraps** | **Rats captured** | **Rats detected** | **ANP (N)** | **APA (%)** |
| --- | --- | --- | --- | --- | --- | --- |
| **Field** | *Apodemus agrarius* | 70897 | 873 | 848 | 20 | 2.36 |
|  | *Rattus losea* |  | 915 | 908 | 22 | 2.42 |
|  | *Suncus murinus* |  | 140 | 138 | 1 | 0.72 |
|  | *Niviventer niviventer* |  | 11 | 11 | 0 | 0 |
|  | *Rattus tanezumi* |  | 8 | 8 | 0 | 0 |
|  | *Rattus norvegicus* |  | 10 | 10 | 0 | 0 |
|  | *Mus musculus* |  | 0 | 0 | 0 | 0 |
|  | *Total* | 70897 | 1957 | 1923 | 43 | 2.24 |
| **Residential** | *Apodemus agrarius* | 71256 | 0 | 0 | 0 | 0 |
|  | *Rattus losea* |  | 11 | 9 | 0 | 0 |
|  | *Suncus murinus* |  | 4 | 4 | 0 | 0 |
|  | *Niviventer niviventer* |  | 5 | 5 | 0 | 0 |
|  | *Rattus tanezumi* |  | 22 | 18 | 0 | 0 |
|  | *Rattus norvegicus* |  | 1679 | 1665 | 30 | 1.8 |
|  | *Mus musculus* |  | 255 | 253 | 5 | 1.98 |
|  | *Total* | 71256 | 1976 | 1954 | 35 | 1.79 |

APN: Antigen positive number; APA: Antigen positive rate.

**Figure 2. The trends of IRV in Gaoan county from 2005 to 2021.** **fitted curve was estimated by loess regression.**


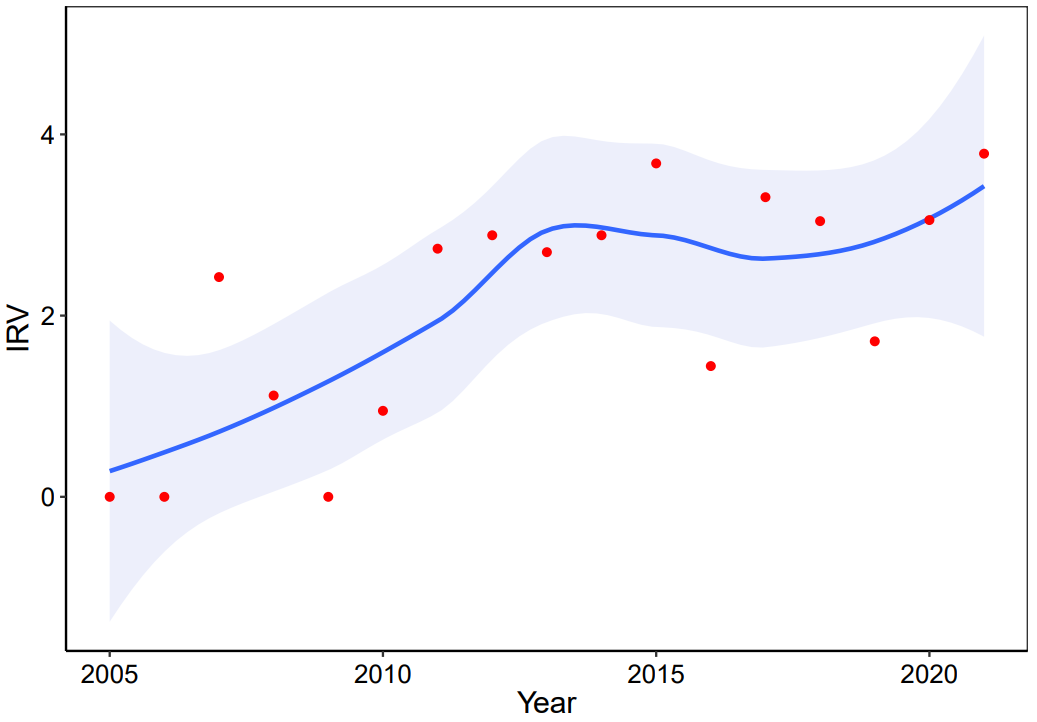


**Table 3. Data collection of Hantavirus strain sequences in Jiangxi**

| Strain | Species | Year | Source | GenBank ID | | |
| --- | --- | --- | --- | --- | --- | --- |
|  |  |  |  | S | M | L |
| L99 | SEOV | 1986 | Rattus norvegicus | AF288299 | AF288298 | \ |
| AY133/2011 | HTNV | 2011 | Apodemus agrarius | KY807166 | KY817594 | / |
| GA21/2011 | HTNV | 2011 | Mus musculus | KY807167 | KY817597 | / |
| GA13/2011 | HTNV | 2011 | Mus musculus | KY807168 | KY817595 | / |
| XJ2/2011 | SEOV | 2011 | Rattus norvegicus | KY80716 | KY817598 | / |
| XJ5/2011 | SEOV | 2011 | Rattus norvegicus | KY807170 | KY817599 | / |
| SG42/2011 | SEOV | 2011 | Apodemus agrarius | KY807171 | KY817600 | / |
| JiangxiXinjianRn-07-2011 | SEOV | 2011 | Rattus norvegicus | KP859511 | KP859513 | \ |
| JiangxiXinjianRn-09-2011 | SEOV | 2011 | Rattus norvegicus | KP859512 | KP859514 | \ |
| SG40/2012 | HTNV | 2012 | Apodemus agrarius | MF141829 | MF141802 | / |
| GAW130/2012 | HTNV | 2012 | Apodemus agrarius | MF141827 | MF141799 | / |
| AYW123/2012 | HTNV | 2012 | Apodemus agrarius | MF141824 | MF141797 | / |
| AYW124/2012 | HTNV | 2012 | Apodemus agrarius | MF141825 | MF141796 | / |
| AYW127/2012 | HTNV | 2012 | Apodemus agrarius | MF141826 | MF141798 | / |
| AYW114/2014 | HTNV | 2014 | Apodemus agrarius | MF141834 | MF141815 | / |
| AYW117/2014 | HTNV | 2014 | Apodemus agrarius | MF141836 | MF141817 | / |
| AYW66/2014 | HTNV | 2014 | Apodemus agrarius | MF141830 | MF141810 | / |
| AYW76/2014 | HTNV | 2014 | Apodemus agrarius | MF141833 | MF141814 | / |
| AYW71/2015 | HTNV | 2015 | Apodemus agrarius | MF141839 | MF141808 | / |
| AYW84/2015 | HTNV | 2015 | Apodemus agrarius | MF141837 | MF141819 | / |
| AYW89-15 | HTNV | 2015 | Apodemus agrarius | KY978755 | KY978756 | KY978757 |
| JXhu04-17 | HTNV | 2017 | Homo sapiens | / | MT782310 | / |
| JXHu10-17 | HTNV | 2017 | Homo sapiens | / | MT782311 | / |
| JXHu14-17 | HTNV | 2017 | Homo sapiens | / | MT782312 | / |
| JXHu27-17 | HTNV | 2017 | Homo sapiens | / | MT782313 | / |
| JXHu33-17 | HTNV | 2017 | Homo sapiens | / | MT782314 | / |
| JXHu39-17 | HTNV | 2017 | Homo sapiens | / | MT782316 | / |
| JXHu46-17 | HTNV | 2017 | Homo sapiens | / | MT782317 | / |
| JXHu47-17 | HTNV | 2017 | Homo sapiens | / | MT782318 | / |
| JXHu49-17 | HTNV | 2017 | Homo sapiens | / | MT782319 | / |
| JXHu50-17 | HTNV | 2017 | Homo sapiens | / | MT782320 | / |
| JXHu51-17 | HTNV | 2017 | Homo sapiens | / | MT782315 | / |
| GAW30/2021 | SEOV | 2021 | Rattus norvegicus | MZ504237 | MZ504239 | MZ504241 |
| GAW50/2021 | SEOV | 2021 | Rattus losea | MZ504238 | MZ504240 | \ |
| "/"no sequences or sequences are not available, "\"sequences are not used in this paper | | | | | | |

**Table 4. Summary of available HV strains in the surrounding provinces of Jiangxi and some other representative reference sequences**

| Strain | Collection site and date | Host | GenBank ID | | |
| --- | --- | --- | --- | --- | --- |
|  |  |  | S | M | L |
| Shashi11 | Hubei/2017 | Apodemus agrarius | MN608061 | MN608071 | / |
| Xiantao92 | Hubei/2012 | Apodemus agrarius | MN608097 | MN608104 | / |
| HV004 | Hubei/ NA | Apodemus agrarius | JQ083395 | JQ083394 | / |
| HV114 | Hubei/1987 | Homo sapiens | AB027110 | L08753 | / |
| HubeiHu02 | Hubei/2002 | Homo sapiens | JQ665905 | JQ665881 | / |
| WuhanRf02 | Hubei/2009 | Rattus flavipectus | JQ665912 | JQ665888 | / |
| WuhanRn10 | Hubei/2009 | Rattus norvegicus | JQ665920 | JQ665896 | / |
| WuhanMm13 | Hubei/2011 | Mus musculus | JQ665910 | JQ665886 | / |
| Hu | Hubei/ NA | Homo sapiens | AB027111 | AB027077 |  |
| SMA029 | Hunan/ NA | Mus musculus | EF210132 | EF210126 | / |
| XXA039 | Hunan/ NA | Rattus norvegicus | EF210136 | EF210130 | / |
| Chen | Anhui/NA | Homo sapiens | AB027101 | / | / |
| FJ35 | Fujian/ 2020 | Rattus norvegicus | MW449190 | MW449189 | / |
| FJ36 | Fujian/ 2020 | Rattus norvegicus | MW449193 | MW449192 | / |
| Fj372/2013 | Fujian/2013 | Suncus murinus | KP645198 | KP645197 | / |
| YN45 | Guangdong/2017 | Rattus norvegicus | MZ031963 | MZ031955 | / |
| XM47 | Guangdong/2015 | Rattus norvegicus | MZ031962 | MZ031954 | / |
| GZ45 | Guangdong/2014 | Rattus norvegicus | MZ031961 | MZ031953 | / |
| MM23 | Guangdong/2017 | Rattus norvegicus | MZ031960 | MZ031952 | / |
| Z10 | Zhejiang/1980s | Homo sapiens | AF184987 | AF143675 | AF189155 |
| ZLS6-11 | Zhejiang/2007 | Apodemus agrarius | FJ753397 | FJ753396 | / |
| ZLS-12 | Zhejiang/2007 | Apodemus agrarius | FJ753396 | FJ753397 | / |
| Z5 | Zhejiang/ NA | Apodemus agrarius | EF103195 | EU074224 | / |
| Z37 | Zhejiang/1980s | Rattus norvegicus | AF187082 | AF187081 | AF285266 |
| ZT71 | Zhejiang/ 2003 | Rattus norvegicus | AY750171 | EF117248 | / |
| ZT10 | Zhejiang/ 2004 | Microtus fortis | AY766368 | DQ159911 | / |
| Gou3 | Zhejiang/ 1983 | Rattus norvegicus | AF184988 | AF14597 | / |
| LongquanAa-08-157 | Zhejiang/2008 | Apodemus agrarius | JQ912698 | JQ912804 | / |
| LongquanAa-10-98 | Zhejiang/2010 | Apodemus agrarius | JQ912760 | JQ912866 | / |
| 84Fli1987 | Shanxi/ NA | Homo sapiens | AF366568 | AF366569 | / |
| Q32 | Guizhou/ NA | Apodemus agrarius | AB027097 | AB027061 | / |
| CGAa31P9 | Guizhou/2007 | Apodemus agrarius | EF990910 | EF990924 | / |
| CGRn15 | Guizhou/NA | Rattus norvegicus | EU363810 | EU363814 | / |
| SN7 | Shichuan/2000 | Rattus norvegicus | AF288657 | AF288656 | / |
| 93HBX12 | Hebei/ NA | Rattus norvegicus | EF192308 | / | / |
| Rod/2016/CD/10/Gc | Hebei/2016 | Rattus norvegicus | / | MK340857 | / |
| Rod/2012/QHD/9/Gc | Hebei/2012 | Rattus norvegicus | / | MK340829 | / |
| SD201 | Shandong/2006 | Rattus norvegicus | GQ279385 | / | / |
| JUN5-14 | Shandong/2004 | Rattus norvegicus | DQ217791 | DQ217790 | / |
| YZG-Changchun | Jilin/2006 | Mus norvegicus albus | EF536376 | / |  |
| [Gongzhuling58](https://www.ncbi.nlm.nih.gov/nuccore/KF745944.1) | Jilin/2009 | Rattus norvegicus | KF745944 | KF745934 |  |
| DN2 | Heilongjiang/2014 | Rattus norvegicus | KX289954 | KX289953 |  |
| 76-118 | South Korea /1976 | Apodemus agrarius | KT885049 | KT885048 |  |
| 80-39 | South Korea/ NA | Rattus norvegicus | AY273791 | S47716 | X56492 |
| DPRK08 | North Korea/2011 | Rattus norvegicus | JX853576 | JX853575 | / |
| SR-11 | Japan/ NA | Rattus norvegicus | M34882 | M34881 | / |
| IR461 | Belgium/ NA | laboratory-acquired infection | AF329388 | AF458104 | / |
| TCH | American/1984 | Rattus norvegicus | KU204960 | KU204959 | / |
| "/"sequences are not avilable or are not used in this paper; NA: not available in Genebank. | | | | | |

**Table 5. Comparative analysis of genome of AYW 89-15 strain with HTNV standard strain (76-118) and vaccine strain (Z10)**

| Strain | Nucleotides | | Amino acid | |
| --- | --- | --- | --- | --- |
|  | 76-118 | Z10 | 76-118 | Z10 |
| AYW89-15 | 86.5/84.5/83.5* | 86.6/84.1/83.4* | 96.7/96.4/97.0* | 97.6/95.3/97.3* |

Note: *S/M/L

**Table 6. Comparative analysis of genome of GAW30/ 2021 strain, SEOV standard strain (80-39) and vaccine strain (Z37)**

| Strain | Nucleotides | | Amino acid | |
| --- | --- | --- | --- | --- |
|  | 80-39 | Z37 | 80-39 | Z37 |
| GAW30/2021 | 89.4/84.7/84.5* | 88.6/84.8/84.5* | 99.5/96.5/97.4* | 98.8/97.0/97.6* |

Note: *S/M/L
